# Modeling vulnerability and intervention targets in the Borderline Personality Disorder system: A network analysis of *in silico* and *in vivo* interventions

**DOI:** 10.1371/journal.pone.0289101

**Published:** 2023-07-31

**Authors:** Said Jiménez, Iván Arango de Montis, Eduardo A. Garza-Villarreal

**Affiliations:** 1 Departamento de Psicología, Tecnológico de Monterrey, Ciudad de México, México; 2 Unidad de Investigación en Medicina Basada en Evidencias, Hospital Infantil de México Federico Gómez, Ciudad de México, México; 3 Clínica de Trastorno Límite de Personalidad, Instituto Nacional de Psiquiatría Ramón de la Fuente Muñiz, Ciudad de México, México; 4 Instituto de Neurobiología, Universidad Nacional Autónoma de México Campus Juriquilla, Querétaro, México; Medical University of Vienna, AUSTRIA

## Abstract

Modeling psychopathology as a complex dynamic system represents Borderline Personality Disorder (BPD) as a constellation of symptoms (e.g., nodes) that feedback and self-sustain each other shaping a network structure. Through *in silico* interventions, we simulated the evolution of the BPD system by manipulating: 1) the connectivity strength between nodes (i.e., vulnerability), 2) the external disturbances (i.e., stress) and 3) the predisposition of symptoms to manifest. Similarly, using network analysis we evaluated the effect of an *in vivo* group psychotherapy to detect the symptoms modified by the intervention. We found that a network with greater connectivity strength between nodes (more vulnerable) showed a higher number of activated symptoms than networks with less strength connectivity. We also found that increases in stress affected more vulnerable networks compared to less vulnerable ones, while decreases in stress revealed a hysteresis effect in the most strongly connected networks. The *in silico* intervention to symptom alleviation revealed the relevance of nodes related to difficulty in anger regulation, nodes which were also detected as impacted by the *in vivo* intervention. The complex systems methodology is an alternative to the common cause model with which research has approached the BPD phenomenon.

## Introduction

Psychopathology network theory is a recent proposal for the conceptualization and modeling of mental health problems [1, 2]. The core of the theory is that the symptoms of mental disorders, which constitute the biopsychosocial problems characteristic of psychiatric diagnoses, are mutually caused [1, 3]. For example, in a person with Borderline Personality Disorder (BPD), which is characterized by instability in interpersonal relationships, self-image, and affections, as well as impulsivity that is accentuated at the beginning of adulthood and occurs in various contexts [4], a relationship breakup event can trigger efforts to avoid abandonment, which can range from excessive attachment to the partner, to threats, psychological manipulation, or self-harm. In turn, these problems can be accompanied by very intense emotional reactions (i.e., anger) that influence the manifestation of extreme problematic behaviors (i.e., substance use), and which could end in a suicidal attempt (for example, breaking up → avoid abandonment → anger → substance use → suicide attempt). Due to the multivariate nature of mental disorders and the multiple interconnections between symptoms, the psychopathology network theory proposes that their components and association patterns can be studied from network structures, in which the nodes constitute the symptoms of the disorder and the edges represent bivariate relationships between symptoms, which are statistically controlled by other variables in the network [1]. The network structure allows mental disorders to be conceptualized as a symptoms constellation that feedback and self-sustain each other, which contrasts with the traditional perspective of psychopathology that considers symptoms as manifestations of a common latent cause (e.g., self-harm, impulsivity, emotional dysregulation -among other problems- occur because of BPD).

The study of psychopathology as networks has advantages over the common latent cause model. Probably the most important being that it allows psychopathology to be characterized at the systems level [2], which implies that instead of reducing the study of the phenomenon by analyzing its components, the overall functioning of the system can be studied, as well as its topology or structure. This raises the possibility of investigating from a new perspective different phenomena such as vulnerability, intervention simulation, and resilience, among others [5–7]. A case that exemplifies the advantages of characterizing psychopathology as a *complex* and *dynamic* system is Major Depressive Disorder (MDD). MDD can be identified as *complex* because the outcomes of the initial configuration of relationships between symptoms are not usually identifiable if the disorder symptoms are investigated on an individual basis; and *dynamic* because interactions in the symptom network evolve in individuals over time [5]. Cramer’s study simulated the within-subject dynamics in the symptom network of MDD, to identify the effect of two *in silico* interventions (performed via computer simulation): 1) manipulating the network architecture in terms of the connectivity strength between nodes that represent symptoms of the disorder (network vulnerability), and 2) exposing the network to external forces that disturb its dynamics such as stress. The study found that individuals with greater strength in the connection between symptoms have more vulnerability to developing MDD. Likewise, a vulnerable network was more likely to end up in a depressed state compared to a less vulnerable network (with less connection strength between symptoms) when it was disturbed by external influences such as stress [5]. The term *in silico* interventions refers to the use of computer simulations to model the behavior of a system (e.g., the BPD symptom network), and explore the potential effects of intervening in a controlled way on its components (e.g., what would happen to the BPD network if we modified the strength of the relationship between its symptoms?). *In silico* interventions require the formalization (mathematical and/or computational) of the components and relationships that make up the system under study (e.g., formalization of a BPD network theory). Formalization limits the vagueness and ambiguity of verbal explanations of phenomena and makes it possible to accurately deduce the behavior implied by the theory. Many researchers today have proposed that the formalization of theories on mental disorders (which allows *in silico* interventions) is an essential requirement to achieve the aims of explaining, predicting, and controlling these problems [8–10].

Another psychiatric disorder in which the network methodology has been implemented is BPD. To date, research on networks and BPD has focused mainly on exploring the network topology, through the calculation of centrality indices, among which the *strength* measure of the node stands out, which consists of adding the absolute weights of the connections for each node [1]. Using this metric, network studies have found that affective instability and identity problems are the nodes with the highest *strength* in the BPD symptoms network [11–14]. This partly coincides with the Dialectical Behavioral Therapy (DBT) theoretical model, which conceives the disorder as a pervasive problem of emotional dysregulation, which occurs through the transaction between the individual emotional vulnerability and the invalidating social environment [15]. Self-functioning is also related to the psychodynamic perspective of personality organization or structure, which through the lens of the object relations theory has integrated developmental, neurobiological, and attachment theory findings for the understanding of self and interpersonal functioning [16]. Although there are other metrics to investigate the network centrality, such as *closeness*, *betweenness*, or *degree*, these measures are based on the structure of the network and do not explicitly consider its *dynamics*, a current proposal to take this dimension into account is the use of *in silico* interventions to alter network characteristics, for example systematically decreasing (or increasing) the predisposition of a symptom to manifest, would allow studying the projected effect of the symptom on network behavior [17]. With this methodology it is possible to investigate what would happen in the system if a specific symptom were intervened, that is if there would be an effect on the total sum of symptoms activated in the network after alleviating or aggravating a certain symptom; which could give the possibility of identifying potential treatment or prevention targets in the real clinical context (*in vivo* interventions).

To our knowledge, none of the aforementioned simulation methods have been implemented for BPD so far, so the aim of the present study was to implement simulations (*in silico* interventions) to model vulnerability, stress response, and potential intervention targets in the BPD symptom network. In addition, DBT is known to be an effective intervention for BPD [18], however, it is unknown which of the symptoms’ network the components of this therapy influence. To validate the results of *in silico* interventions, we investigated the correspondence between the *in silico* intervention results (alleviating or aggravating specific nodes) in the BPD symptom network and the symptoms impacted by an *in vivo* DBT intervention in patients. Specifically, the four aims of our study are listed below:

1. Examine through simulations the impact of manipulating vulnerability (connectivity between symptoms) on the behavior of a within-subject network of BPD. To determine if a system with greater connectivity between symptoms would more easily end up in a severe state of symptomatology of the disorder.
2. Explore the effect of manipulating (increasing or decreasing) stress on the BPD symptom network. To assess the dynamics of the change from a state of little or no symptoms severity to one of greater severity.
3. Evaluate the projected effect of simulated interventions (*in silico*) of aggravating and alleviating specific symptoms, on the global behavior of the BPD symptoms network, to determine potential *in vivo* intervention targets.
4. Assess the effect of the DBT skills training group through network analysis to determine the symptoms directly related to the intervention and explore the possible correspondence between results of *in silico* and *in vivo* interventions.

## Materials and methods

### Data

For the first three aims, we used the open data from von Klipstein [19], which includes 683 subjects diagnosed with BPD from four different longitudinal studies that evaluated various treatments [20–24]. Although the studies made multiple measurements during different periods, the four papers evaluated BPD symptomatology during the baseline with the semi-structured interview BPDSI-IV, which consists of 70 items that record the 9 BPD symptoms defined by the DSM-IV on an 11-point scale, ranging from “never” to “daily”. The BPDSI-IV has good reliability and validity indices [25, 26]. Only the baseline measurements of the 683 patients in the 70 interview questions were used, and a classification of 1 was made for responses with a score equal to or greater than the average for that symptom and 0 for responses below that threshold. Lost data were imputed with the median of the variable and were subsequently binarized with the described criteria.

For the fourth aim, data from 127 patients with BPD recruited at the Instituto Nacional de Psiquiatría Ramón de la Fuente Muñiz in Mexico City were used. The patients underwent the DBT skills training group and the effect of the intervention was recorded with the self-report instrument *Borderline of Severity Evaluation over Time* (BEST) [27] applied before and after the intervention. More details about the sample and the intervention can be found in another publication [28]. The BEST test consists of 15 multiple-choice questions, the first 12 assess negative thoughts, feelings, and behaviors related to BPD symptoms, and their response categories range from Not at all = 1 to Extreme = 5. While the last three questions measure positive behavior that may indicate the effectiveness of the intervention. For the purposes of the statistical analysis carried out in this work, only the answers to the first 12 items were considered, since they are the questions that measure the main BPD symptoms according to the DSM-IV. Missing cases were substituted for the median and responses with no transformations were used for analysis as they were obtained from the participants. Both data sets were acquired with the consent of the participants and all procedures were performed in accordance with the Declaration of Helsinki.

### Simulation 1: Examine the vulnerability of the BPD network

First, a between-subjects network of BPD symptoms was built, with the measurements of the nine characteristic symptoms obtained using the BPDSI-IV. With the empirical parameters estimated in this network, the within-subjects dynamics of the disorder over time were simulated in three individuals (three systems) with different levels of vulnerability. By manipulating the connectivity strength between nodes in the network, an individual with little vulnerability (system with weak connectivity), another with moderate vulnerability (system with medium connectivity), and a third with high vulnerability (system with strong connectivity) were simulated. The simulation started with zero activated symptoms and then evaluated how the system evolved over time assuming that the symptoms can cause each other, for example mood instability (i.e., feeling irritable), can lead to an outburst of anger. The hypothesis is that more vulnerable individuals (systems with greater connectivity) will more easily develop a greater number of activated symptoms than the less vulnerable [5].

#### Empirical parameters

**Fig 1:**
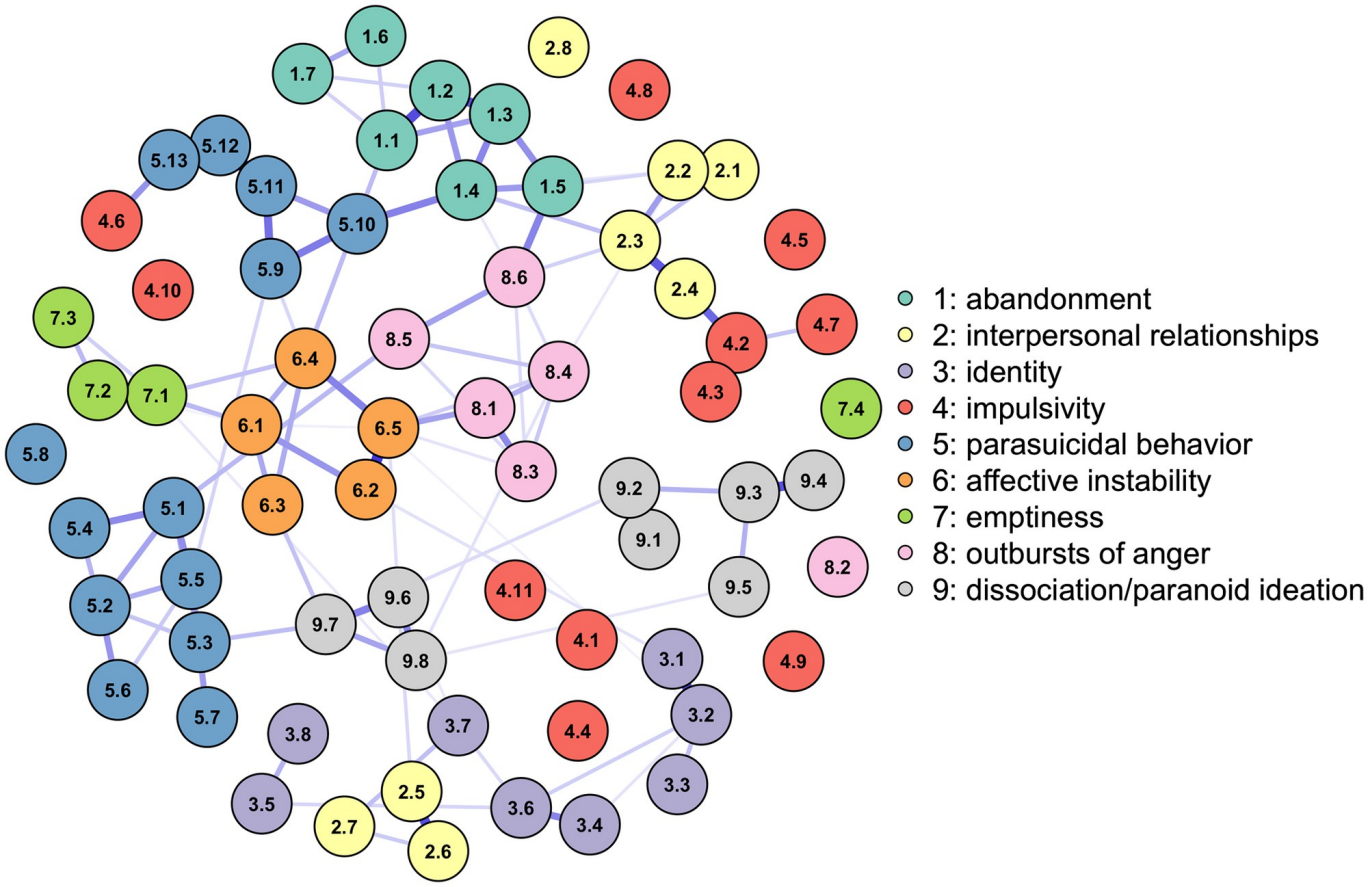
Between-subjects network of Borderline Personality Disorder estimated with the Ising model. The nodes represent the 70 symptoms/sub-symptoms of the BPDSI-IV instrument, and the edges reflect positive dependencies between the symptoms: a tendency between the symptoms to stay in the same state of activation (e.g., both symptoms on). The colors of the nodes reflect the category of the top 9 BPD symptoms to which a symptom belongs. While the edges indicate the strength and direction of the relationship between the symptoms, the darker blue color and the more saturated edges indicate a stronger dependency between nodes.

With the Ising model developed for binary data and implemented in the R package *IsingFit* [29], the parameters of an inter-individual network of the 70 symptoms and sub-symptoms measured with the BPDSI-IV were estimated in a sample of 683 patients (Fig 1). The Ising model estimates two sets of parameters: *thresholds* and *weights* [30]. The *thresholds* determine the disposition of a symptom to be on or off, positive values indicate the disposition to be activated when the other symptoms are absent, while negative values indicate the disposition to be deactivated. And the *weights* reflect the connection between two network symptoms, higher values indicate that the symptoms prefer to be in the same state, while lower values indicate that they prefer opposite states, and a weight of zero indicates that there is no connection between the symptoms [5]. The parameters of the Ising model are estimated using logistic regression analysis, in which the probability that a symptom is activated depends on all the other symptoms. The *thresholds* correspond to the intercepts of the logistic model and the *weights* are the regression coefficients that estimate the relationship between a specific symptom and another when the other symptoms in the model are deactivated (i.e., when they are zero). The parameters are regularized through the *l*1 penalty, which implies that the coefficients can take a value of zero depending on the size of the hyperparameter *λ*, which for this analysis was selected with the Extended Bayesian Information Criterion (EBIC). The stability of the network and its parameters was analyzed with 4000 repetitions of the *case-dropping bootstrap* technique and the calculation of the Correlation Stability coefficient (CS-coefficient) [31].

#### Formal model of BPD dynamics

The model presented in simulations 1 and 2 is an adaptation of Cramer’s proposal for MDD but implemented for Borderline Personality Disorder [5]. The model assumes that 1) symptoms can be active or inactive, 2) activation of symptoms occurs over time (i.e., first the patient may feel empty, and then this may lead to self-harm), and 3) the activation of a symptom depends on the activity of neighboring symptoms. Regarding the validity of the assumptions underlying the formal model, it can be mentioned that they are in tune with the principles of the network theory of mental disorders [1], and with the assumptions of the Ising statistical model that was used to obtain the parameters that allow building the network of symptoms [32]. They are also the same assumptions used in Cramer’s original model for MDD [5].

The total activation function formalizes the BPD dynamics in the following expressions:

$$A_{i}^{t}=\sum_{j=1}^{J}{\text{W}}_{i,j}X_{j}^{t-1}$$

The function $A_{i}^{t}$ is the total amount of activation that symptom *i* receives at time *t*, which is defined as the sum of the state of all neighboring symptoms **X** at time *t* − 1 weighted by **W**, which represents the matrix of the *weights* acquired with the Ising model. The term *W*_*i*,*j*_ indicates the logistic regression coefficients between the symptoms *i* and *j* obtained empirically for the *J* = 70 symptoms. The term *X* contains an array of 0’s and 1’s that indicate which symptoms are (de)activated at the prior moment (*t* − 1) to the calculation of the total amount of activation.

To obtain the probability that a symptom becomes active at a given time, we use the following logistic function:

$$P\left(X_{i}^{t}=1\right)=\frac{1}{1+e^{\left(b_{i}-A_{i}^{t}\right)}}$$

The function indicates that the probability that a symptom *i* is active at time *t* depends on the difference between the total activation of neighboring symptoms and the *threshold* of symptom *i*. If the received activation ($A_{i}^{t}$) is greater than the *threshold* of the symptom (*b*_*i*_), the probability of the symptom being activated increases, while if the received activation is lower than the *threshold*, this probability decreases. The *threshold* of the symptom **b**_**i**_ is the absolute value of the empirically estimated parameter, that is, the intercept of the logistic regression model. In short, a symptom that has little resistance to activation by neighboring symptoms is more likely to be active.

#### Simulation setup

To investigate the vulnerability in the system, the connectivity between the symptoms was manipulated through the parameter *c* that modulates the amount of activation that a symptom can receive. The *total modified activation function* is shown below:

$$A_{i}^{t}=\sum_{j=1}^{J}c{\text{W}}_{i,j}X_{j}^{t-1}$$

The *c* parameter represents the factor by which the network connectivity is modulated, values less than 1 indicate weakening, and values greater than 1 indicate a strengthening of the connections. As a starting point, we consider all the symptoms deactivated and we simulate 1500 points in time. In each simulation, the state of the network was monitored by calculating the sum of activated symptoms as well as the probability of activation of each symptom. For the next time point the status of each symptom, whether it was active or inactive, was obtained using the probabilities from the previous iteration. The sum of activated symptoms was a measure of the entire system *D* = ∑(*X*). The higher the sum the more *distressed* the system was considered.

### Simulation 2: Investigate the influence of external stressors

The purpose of the second simulation is to assess the responses of BPD systems that vary in the degree of vulnerability (connectivity) to stress manipulation, in order to record the dynamics of change from a state of little or no symptom severity to one of greater severity.

#### Simulation setup

For the second simulation, we used the same configuration as the first, however, the parameter $S_{i}^{t}$ was included, which represents systematic increases (or decreases) in the stress of the network, the higher the value of $S_{i}^{t}$ is the greater the value of the total activation function.

$$A_{i}^{t}=\sum_{j=1}^{J}c{\text{W}}_{i,j}X_{j}^{t-1}+S_{i}^{t}$$

Therefore, the *total activation function* indicates the sum of the activation received by a certain symptom, which, as presented in simulation 1, can vary depending on the level of vulnerability of the network. For this simulation, four levels of connectivity were modified to represent within-subjects networks with weak (*c* = 0.2), medium (*c* = 1.1), strong (*c* = 3.0), and extreme (*c* = 10.0) vulnerability. We simulated 10,000-time points where the parameter $S_{i}^{t}$ was included with gradual changes from -15 to 15 to represent *increase* in stress, and over a range of 15 to -15 to represent *decrease* in stress. In each simulation, the global state of the network was obtained with the sum of all the symptoms activated (*D* = ∑(*X*)), and the impact of the parameter *S* on the behavior of the system was measured with the calculation of the average number of active symptoms at a given point in time *t*. Averages were obtained at intervals of approximately 0.15 of change in stress level and *increases* and *decreases* were differentially quantified.

### Simulation 3: Evaluate the effects of intervening specific nodes

The first two simulations are based on altering the connectivity of the system (*c* **W**) to represent individuals with different levels of vulnerability, on the other hand, the third simulation modifies the disposition of individual symptoms to manifest without affecting connectivity. In other words, the third simulation decreases or increases the *threshold* of each symptom to emulate what would happen in the system if a specific symptom were intervened in one of two directions: alleviating or aggravating. In general, therapeutic interventions do not seek to increase symptoms; however, this exercise would make it possible to identify important nodes in the network that could be potential targets for intervention or prevention of BPD.

#### Simulation setup

The simulation was run with the *nodeIdentifyR* (NIRA) algorithm, implemented with the R package of the same name [17]. The NIRA performs multiple simulations in which interventions are administered by systematically modifying the *thresholds* of each symptom estimated in the original network model (Fig 1). The algorithm generates 5,000 simulated patients for whom symptom measurement was performed **after** performing the intervention. Implemented to the [19] data, this method generated 71 X 5000 observations, once with the original network parameters and 70 times for each alteration of the individual *thresholds*. Two types of interventions were carried out: 1) **alleviating** interventions decrease the disposition of a symptom to manifest by subtracting an amount from the original value of its *threshold* parameter and 2) **aggravating** interventions increase the disposition of a symptom to manifest itself by adding a quantity to the original value of the symptom’s *threshold* parameter. In the present simulation, we obtained the standard deviation of all *thresholds* and decided to subtract it twice for the alleviating intervention and add it twice for the aggravating intervention [17]. After the intervention of each symptom, the average number of activated symptoms was calculated as a measure of the impact that specific disturbances had on the global BPD system. And the NIRA score was calculated as the absolute difference between the average of the scores without intervention and the average after the interventions. The node with the greatest difference is the symptom with the greatest projected effect on the behavior of the network.

## Results

### Results of simulation 1: Vulnerability of the BPD network

**Fig 2:**
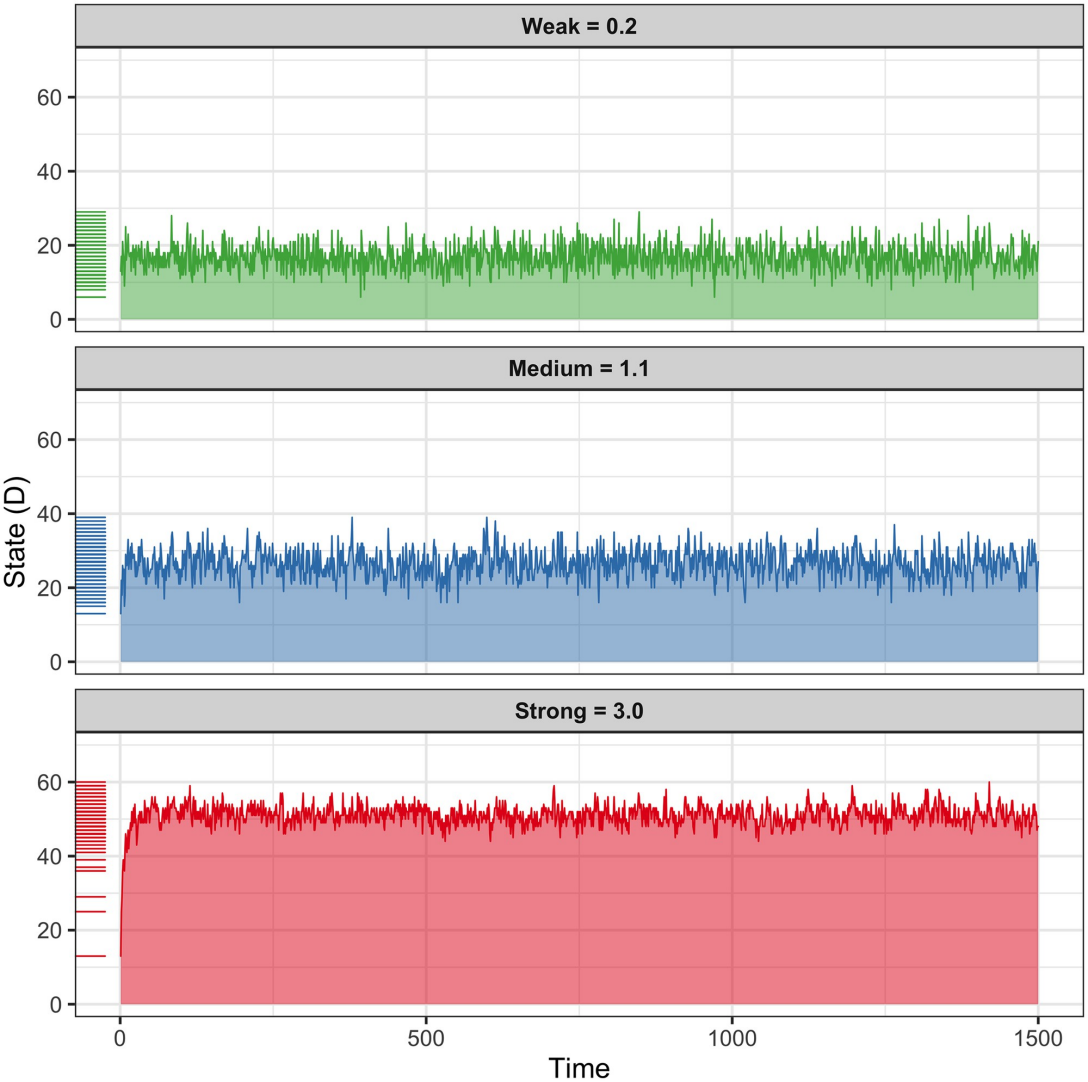
Within-subjects vulnerability to BPD. The activation dynamics over time of three simulated within-subjects networks are shown, which suffered from the manipulation of the empirically estimated connectivity parameters. The three panels show the behavior dynamics in the network under different levels of vulnerability, the X axis represents the time, and the Y axis the number of activated symptoms (D). The top panel shows activity when connectivity is weak, representing a system with low vulnerability, the middle panel shows moderate connectivity and vulnerability scenarios, and the bottom panel shows strong connectivity and vulnerability. The higher the time series indicates, the more symptoms are activated.

The hypothesis of this simulation was that increasing vulnerability, by increasing the connectivity of the BPD within-subjects network would more easily increase the number of symptoms activated in the system, causing a generalized state of distress (D). The results are consistent with the hypothesis, the strong connectivity system (*c* = 3.0) had an average of 51 points out of 70, 72.9% activation of the network, with scores ranging mainly between 40 and 60 points of the state of distress (D). In the lower part of (Fig 2), the strong connectivity system quickly reached its average activity, and once at that level, its behavior was *stationary* (without drastic changes in its mean and variance, nor trend or periodicity). The middle part of Fig 2 shows the results for the moderate connectivity system (*c* = 1.1), which had an average activity of 26.5 out of 70, 37.9% network activation; the values of this simulation were in a range of 13 to 39 and mainly stayed between 20 and 40 points. Finally, the upper part of Fig 2 shows the state of the network with weak connectivity (*c* = 0.2), which had an average activity of 16.8 points out of 70 possible, with 24% network activation. The scores for this simulation ranged from 6 to 29 and remained mostly between 10 and 25. It should be noted that in none of the 3 simulated cases did the network activation score reach its minimum (*D* = 0) or maximum value (*D* = 70). However, a wide range of activation did occur from 6 and up to 60 points, 8.6% and 86.7% of total activation of the network respectively. This could suggest that rather than going from *attractor* states (from no symptoms to all symptoms) the state of distress in the network (*D*) is a continuous variable rather than categorical.

### Results of simulation 2: Influence of stress on the system

**Fig 3:**
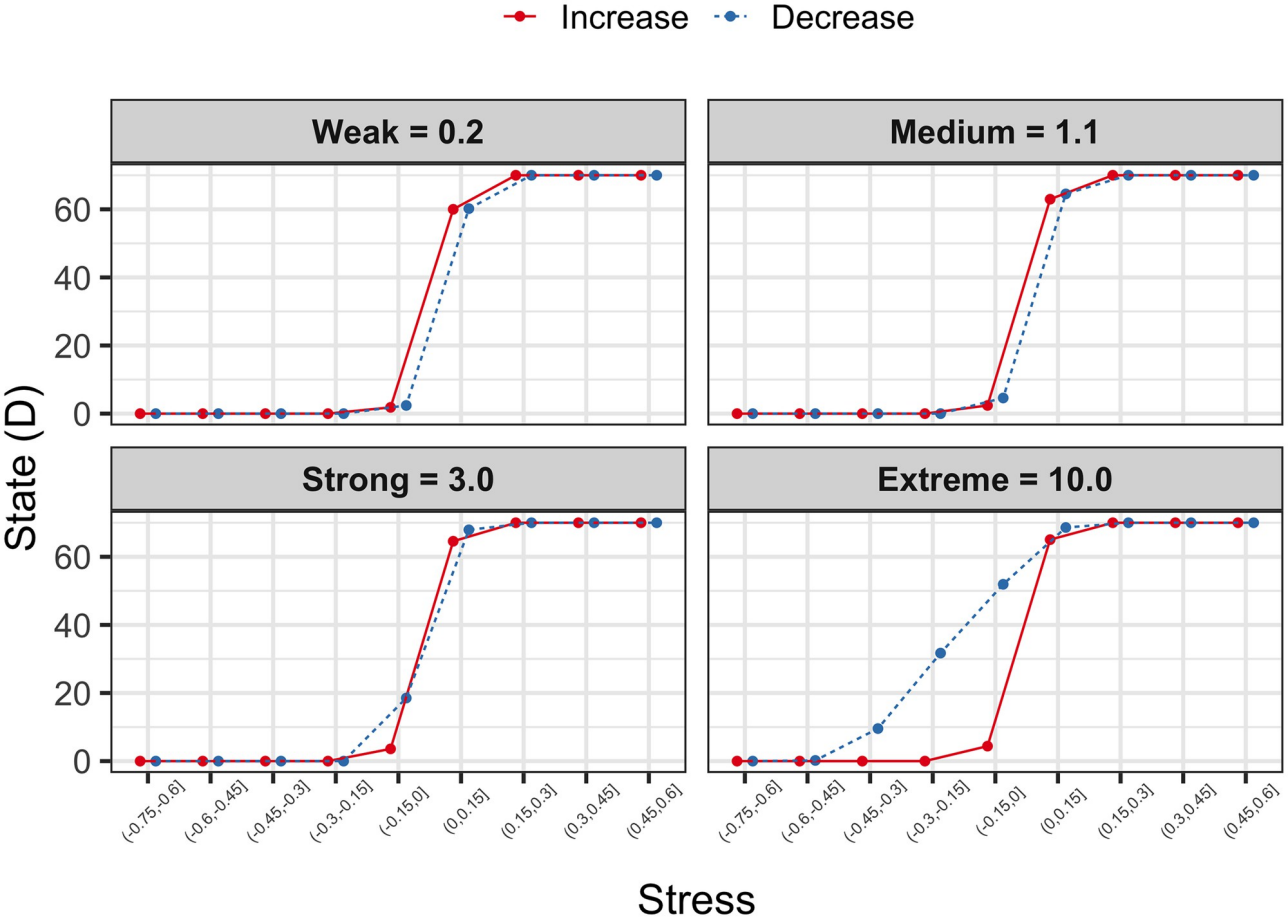
Influence of stress on the BPD system. The results of four simulations in which stress is gradually increased or decreased in systems that vary in their level of vulnerability are shown. The X axis represents the stress that increases or decreases in average intervals of 0.15, and the Y axis shows the state of the network from zero activation D = 0 to full activation D = 70. The red line represents the behavior of the system when faced with increases in stress, while the dotted blue line indicates the decrease in stress. It is worth noting the hysteresis pattern shown in the Strong and Extreme connectivity panel.

For this simulation there was no concrete hypothesis, rather we set out to explore the potential effects of gradually increasing or decreasing stress, on the state of BPD symptom networks that differ in their level of vulnerability. Both increases and decreases in stress in weak or moderate connectivity systems (*c* = 0.2 and *c* = 1.1) had very similar effects on the level of network activation. It is observed that when the stress is negative, the state of the network is practically inactive (*D* = 0), but in the transition from negative to positive stress values, from -.15 to.15, there is a dramatic change in the behavior of the network, which goes from being with zero activation to values of 60 activation points or more (Fig 3, *weak* and *medium* panels), while the reverse pattern exists when it comes to stress reductions (e.g. drastic change of complete to null activation). On the other hand, the simulation of the case with *strong* connectivity reveals an interesting behavior when the stress is reduced, in the range of.15 to -.15 the activation of the network also drops dramatically, however it does not drop until the lowest level of distress, but it is reduced from approximately 70 to 19 points of D and later in the range of values less than -.15 its activity decreases to zero (Fig 3, *strong* panel). This observation led us to simulate a fourth case, a system with extreme vulnerability (*c* = 10.0), which shows great resistance to falling in its activation level, the transition of stress values of on average.15 at -.15 it only roughly reduces the activation of the network from 70 to 51 points, in the next range of -.15 on average the network decays from 51 to 31 points and from 31 to 10 points in the following decrease range, to finish at the level of zero activation up to the stress range of -.45 to -.60 (Fig 3, *extreme* panel). This behavior of resistance to returning to a baseline level of activation even when the stress level in the network has been reduced is similar to that observed in depression systems [5] and has been called *hysteresis*. It is worth emphasizing that this behavior pattern is specific to decreases in stress since increases only show a sharp transition when crossing the zero threshold (Fig 3). Meaning that, even with a decrease in stress, symptoms may resist improvement.

### Results of simulation 3: *In silico* intervention to specific nodes

**Fig 4:**
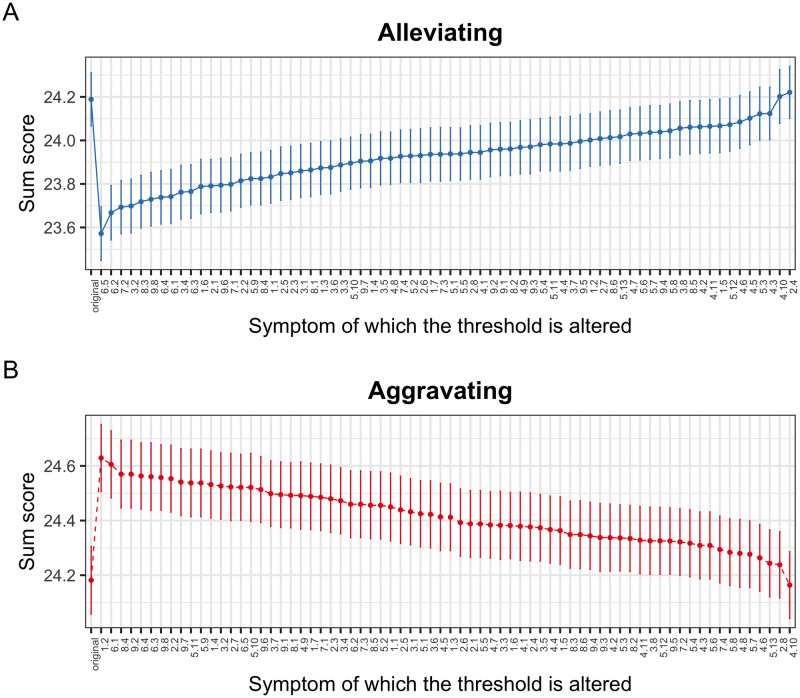
In silico intervention to specific nodes. The projected effects of the simulated interventions (alleviating or aggravating) to specific nodes are shown. The X axis orders the nodes from most to least important in terms of the projected effect on the state of the network, while the Y axis registers the level of activation of the system, the higher the score, the greater the severity of the disorder. Panel A shows the results for the alleviating intervention and panel B for the aggravating intervention.

This simulation has the potential advantage of identifying the projected importance of specific nodes in the BPD symptom network. The symptom *alleviating* intervention identified nodes 6.5 (how often did you notice your mood fluctuate toward feeling angry?), 6.2 (how often did you notice your mood fluctuate toward feeling irritable?), 7.2 (how often did your emptiness or boredom prevent you from doing something you wanted to do?) and 3.2 (To what extend did it happen that the idea of who you are, changed strongly?), as the four symptoms that, when reduced with an intervention, would have the greatest effect on the global state of the network, would allow the system to change from an activation of on average 24.2 to around 23.6 (Fig 4A). While the symptoms *aggravating* intervention identified nodes 1.2 (how often did you act frankly, to prevent someone from leaving you?), 6.1 (how often did you notice your mood fluctuate towards feeling depressed or dejected?), 8.4 (how often were you so angry that no-one could approach you or reason with you?) and 9.2 (how often did your surroundings seem strange or unreal?) as the four symptoms that would have the greatest effect on the network if they were worsened, these nodes would increase the total activation score of the network from 24.2 to approximately 24.6 (Fig 4B).

**Fig 5:**
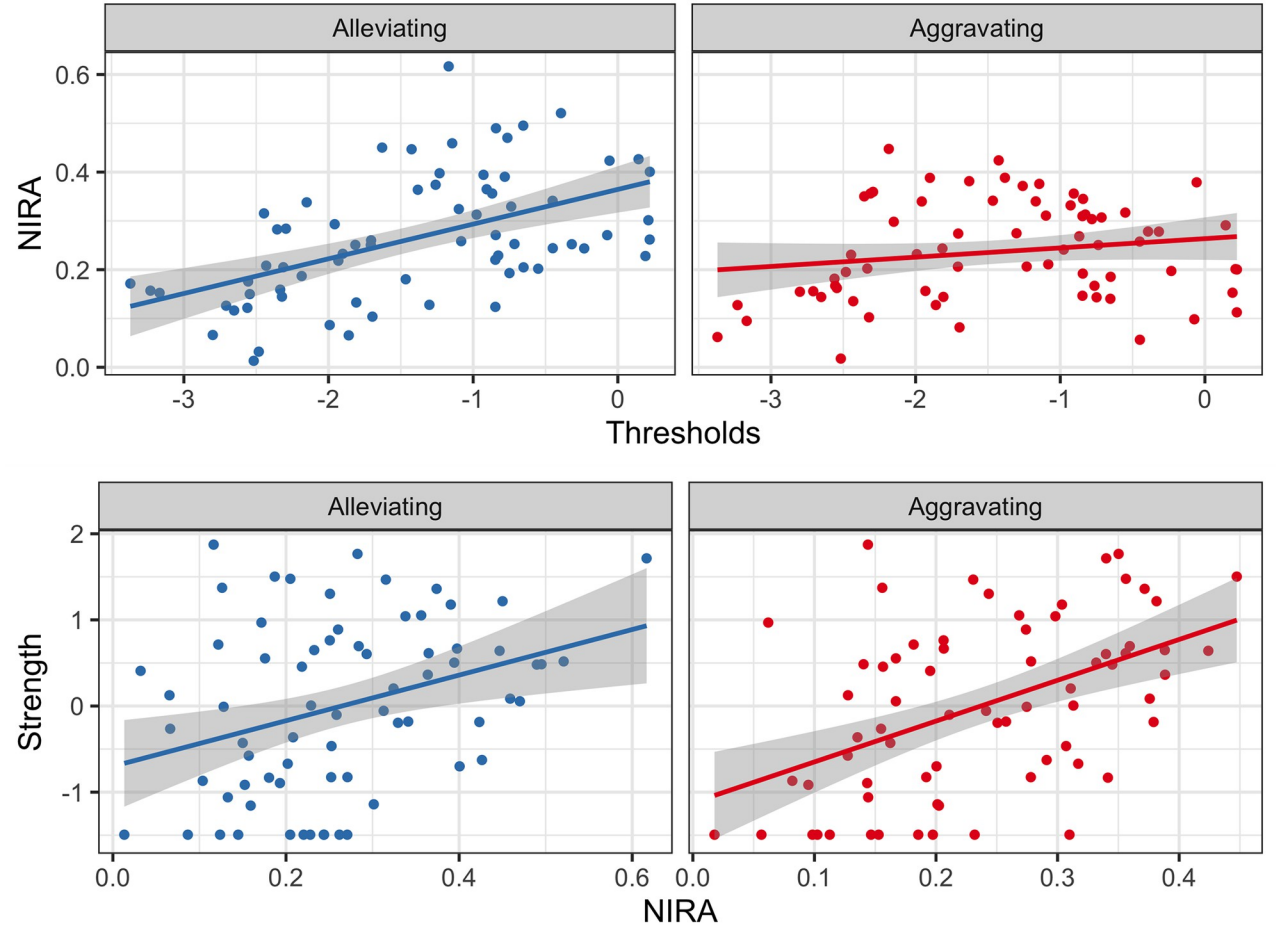
Relationship between the thresholds, the measure of centrality strength and the NIRA. The NIRA is obtained by calculating the absolute difference between the average of the scores without intervention and the average after the interventions. The node with the highest NIRA score is the symptom with the greatest projected effect on the behavior of the network. The correlation of this statistic with the strength centrality measure (bottom) and the thresholds of each symptom (top) are shown.

In the context of *in silico* interventions, it could be hypothesized that the greater the disposition of a node to manifest itself, the greater the projected effect of that node. To identify this relationship, the Pearson correlation between the *thresholds* of the symptoms and the NIRA score was calculated, which is obtained by calculating the absolute difference between the average of the scores without intervention and the average after the interventions, and indicates that the node with the greatest difference (i.e., more NIRA score) is the symptom with the greatest projected effect on the behavior of the network. For the alleviating *in silico* interventions, the correlation between the NIRA and the *threshold* of each symptom was moderate-high, *r* = .52, while for the aggravation interventions, this relationship was low, *r* = .18 (Fig 5 top). Similar to the above, a positive relationship between network centrality measurements and the projected effect of a node would also be expected. The relationship between the *strength* centrality measure and the NIRA score was investigated by calculating the Pearson correlation coefficient for each type of intervention. When the interventions were alleviating, the relationship between *strength* and the NIRA was moderate-low, *r* = .34, while when they were aggravating, the relationship was moderate-high, *r* = .48 (Fig 5 bottom). Therefore, it can be concluded that the *strength* centrality measure can provide similar information to the NIRA, more in the case of aggravating interventions than alleviating. And in the case of the *thresholds* of symptoms, it can be mentioned that particularly for alleviating interventions, this parameter can provide information on the projected effect of a node, but this is not the case for aggravating interventions. These results suggest that there may be key symptoms that if treated would reduce the overall BPD severity by affecting the rest of the symptoms.

### Results of the empirical study: Analysis of the *in vivo* group intervention

**Fig 6:**
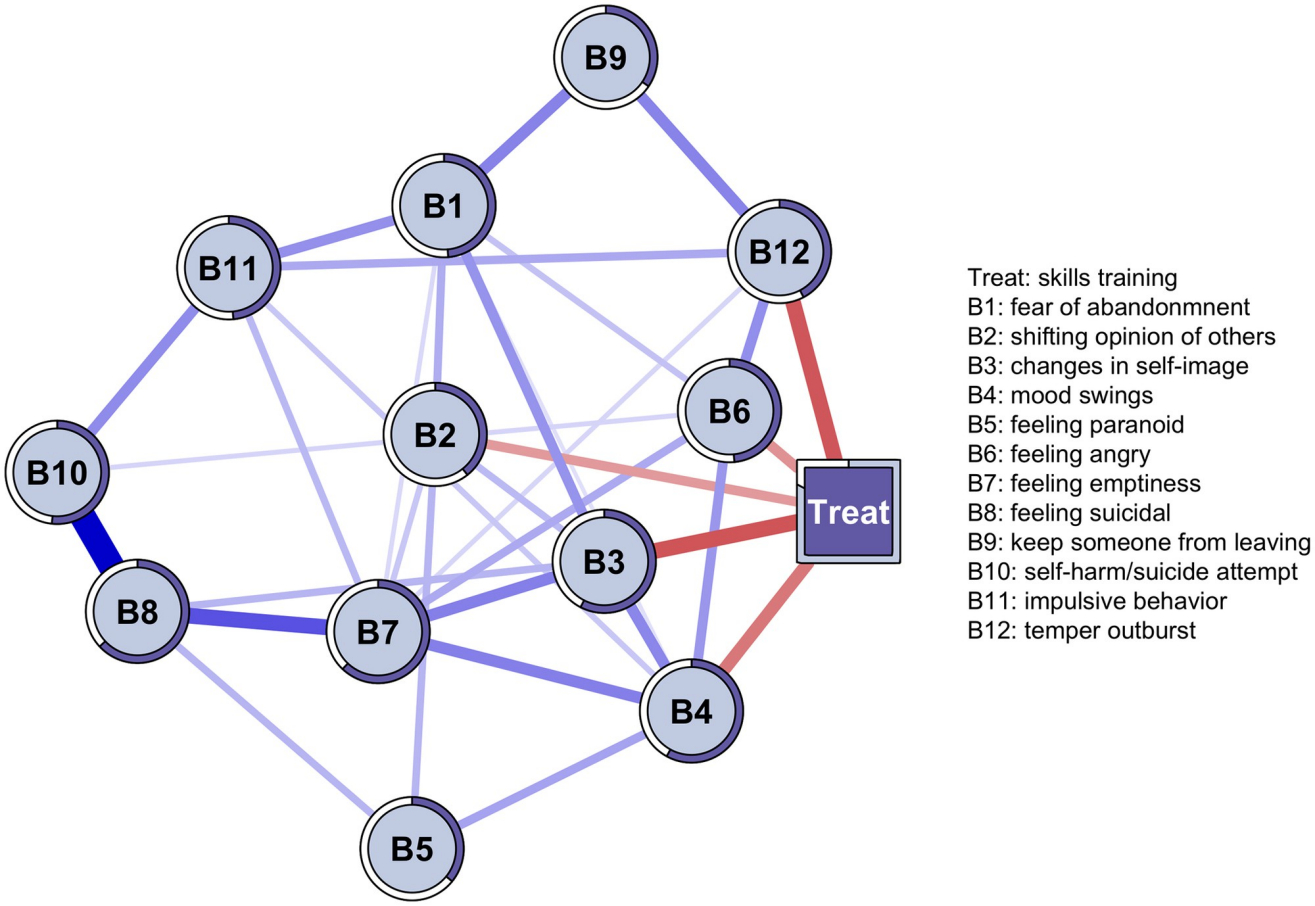
Network obtained with the mixed graphical model to assess the relationship between BPD symptoms and the in vivo intervention. The network shows the dependencies between disorder symptoms (circular nodes) and the intervention (square node). Blue connections indicate positive relationships, while red ones indicate negative relationships. The width and intensity of the edge reflect the strength of the association. The circles that surround each node are predictability measurements, the more circumference is covered reflects the greater predictability. The contour at the square node is also a measure of predictability and indicates correct classification by the other nodes.

The network obtained with the MGM of the 12 items of the BEST instrument that assess the severity of BPD symptoms can be seen in Fig 6. The network reveals negative connections between the presence of the intervention (Treat) and the nodes B12 (temper outburst), B6 (feeling angry), B2 (shifting opinion of others), B3 (changes in self-image), and B4 (mood swings). The strong connections between the treatment and nodes B3 and B12, which have a content relationship with symptoms 3.2 (To what extend did it happen that the idea of who you are, changed strongly?) and 8.4 (how often were you so angry that no-one could approach you or reason with you?) of the BPDSI-IV, identified as relevant symptoms in the *in silico* interventions (Fig 4). Likewise, nodes B4 and B6, associated with the treatment, find parallelism with nodes 6.5 (how often did you notice your mood fluctuate towards feeling angry?) and 6.2 (how often did you notice your mood fluctuate towards feeling irritable?) identified in simulation 3. Other relevant connections due to their strength of relationship are those that occur between nodes B7 (feeling emptiness), B8 (feeling suicidal), and B10 (self-harm/suicide attempt), they are also important because self-harm, suicidal desire, and suicidal attempts are probably the most serious symptoms of the disorder. It is worth noting that node B7 on the feeling of emptiness is also associated with nodes B3 and B4, which measure changes in self-image and mood, and which in turn were strongly related to the presence of the intervention (Fig 6).

Regarding predictability, nodes B3, B4, B7, B8, and B10 showed scores of explained variance *R*^2^ greater than.5, while all the other nodes, with the exception of B2, had *R*^2^ >.4. In the treatment node that is a categorical variable of two levels, the predictability can be evaluated with the correct classification (CC), which was.83. These predictability scores suggest that the network was largely shaped by strong interactions between symptoms and less by other factors. In terms of network stability, the 2500 *bootstrap* replicates revealed stability of the connection between the intervention and node B12 *temper outburst* (mean = −.23, 95% CI = −.50,−.02), as well as the intervention and node B3 *changes in self-image* (mean = −.23, 95% CI = −.50,−.02). The stability of all connections and measures of centrality estimated with *bootstraping* can be reviewed in S1 Appendix. On the other hand, the *case-dropping bootstrap* with 2500 repetitions allowed us to calculate the CS-coefficient, which indicates the maximum percentage of cases that can be discarded to maintain a correlation of 0.7 with at least 95% of the samples [31]. The CS-coefficient for the *edges* was.44, which indicates that their interpretation should be done with caution. For the centrality indices, the CS-coefficient of *strength*, *closeness*, and *betweenness*, was .21, .13, and .05, respectively. Since these centrality measurements did not obtain optimal coefficient values, it was decided not to include or interpret them.

## Discussion

The present study had the aim of implementing three simulations to investigate: 1) vulnerability, 2) the response to stress and 3) the *in silico* intervention to alleviate or aggravate symptoms, in the Borderline Personality Disorder (BPD) system. Another aim was: 4) to analyze the relationship between an *in vivo* group intervention based on Dialectical Behavioral Therapy (DBT) and the BPD symptom network. Regarding the first aim, we found that the within-subjects networks of BPD symptoms were sensitive to the strength of the connections between nodes, such that a network with greater connectivity showed a dynamic of a greater number of activated symptoms in contrast to networks with moderate and low connectivity, thus reflecting the behavior of systems that differ in their degree of vulnerability (the more connections between symptoms the more vulnerable). Notably, regardless of the level of system connectivity, the state of the network never reached its highest or lowest distress value. The fact it did not reach the highest level is understandable since there were nodes that did not show a connection with the rest of the network, so it was not plausible that they would be affected by the activation of the other nodes. However, it was possible that the activation of the system fell completely (*D* = 0), but it was something that did not occur. This observation contrasts with the simulations carried out for MDD where spontaneous remissions of system activity do occur, which have been interpreted as subjects who recover without apparent cause from their depressive state [5]. In the case of BPD, it seems that the activation of the system does not oscillate between *attractor* states (e.g., depressive versus non-depressive), but rather it keeps fluctuating at a level of activation proportional to the vulnerability of the system. In clinical terms, these observations could suggest that, at least in the short term, spontaneous remission is unlikely to occur in BPD (although a 10-year course of BPD is characterized by high remission rates [33]), and that the individual’s level of vulnerability could be a very consistent predictor of the severity of their future psychopathology. However, both interpretations require empirical validation.

Regarding the second aim, the simulation showed that exposing a within-subject BPD network to gradual stress increases causes a sudden fluctuation between opposite states, from zero activity to full system activity, regardless of the level of vulnerability of the network. This occurs specifically in the escalation of stress when the threshold that divides the negative and positive values of the variable is exceeded. When the network is exposed to gradual decreases of stress, an effect of *hysteresis* was observed in networks with strong and extreme vulnerability, which consists of the tendency of the system not to decrease its activation despite having removed the source of stress. This pattern was much more notable in the system with extreme vulnerability compared to the one with strong vulnerability. This observation was consistent with what was detected in simulations for MDD and other systems [5, 34], and in the clinical context, it could be interpreted as residual discomfort that occurs after a highly emotional event. According to Linehan (1993), in the context of BPD, emotional vulnerability is considered to have the components of 1) hypersensitivity and 2) emotional hyperreactivity, and 3) difficulty returning to baseline. The first two indicate that in individuals with BPD, little stimulation is required to trigger an emotion and that when it manifests it does so in great magnitude; while the third point assumes that the emotion has a significant duration and a lot of resistance to fade. The *hysteresis* behavior shown in the systems with the greatest connectivity in our study could correspond to the third component of the emotional vulnerability proposed in Dialectical Behavior Therapy (DBT), which in clinical terms could imply that patients with greater connection strength between their symptoms would also tend to manifest greater difficulty to recover from an emotional experience than patients with less vulnerability in their system.

Regarding our third aim, three of the most important nodes detected in the simulations of *alleviating* interventions were from the affective instability dimension, which content refers to the experience of fluctuations in the emotions of anger and irritability; while for *aggravating* interventions depression was the most important node. According to the biosocial theory of BPD [15, 35], the emotional vulnerability of biological origin and social invalidation are the elements that cause the pervasive emotional dysregulation characteristic of the disorder. However, to our knowledge, the theory does not propose differences in terms of the hierarchy that could have certain emotional states with respect to others, which assumes that all emotional states could be dysregulated in the same way and magnitude. Although the results of our simulation are consistent with biosocial theory in general, our findings suggest greater relevance of certain emotional states over others, in terms of the projected effect that their intervention imposes on the network. In its dimension of emotional instability, the BPDSI-IV questionnaire investigates mood fluctuations towards depression or dejection, irritation, anxiety, despair, and anger, which is why it is noticeable that irritability and anger were detected as the most important nodes in the BPD system in the modality of *alleviating* interventions. It is known that these two emotions belong to the same semantic field and affective dimension and that people are capable of discriminating between their frequency of occurrence despite the fact that they are usually highly correlated [36]. Therefore, the results of the *in silico* intervention could suggest that irritation and anger should have priority for *in vivo* intervention, more than other emotional states such as anxiety, depression, or despair (however, it is worth mentioning that the *aggravating* intervention suggests preventing mood swings towards depression). Another reflection that can be drawn from the results of the *in silico* intervention is that the understanding of emotional vulnerability proposed in the biosocial theory of BPD could be refined in its description of the most relevant affective states to understand the generalized emotional dysregulation in this disorder.

Regarding the fourth aim, the *in vivo* intervention with the DBT skills training group was related to a decrease in outbursts of temper, feelings of anger, and changes in mood. Likewise, it was related to a decrease in instability in the opinion about others and self-image. It should be noted that DBT skills training teaches patients four main skills: 1) mindfulness, 2) emotional regulation, 3) social skills, and 4) distress tolerance and crisis survival [15]. Mindfulness, emotional regulation, and discomfort tolerance skills mainly coincide with the nodes impacted by the intervention (e.g., emotional regulation skills are likely to impact changes in mood), hence the interactions between nodes that were detected with the analysis were consistent with what is theoretically expected. Mechanisms of change that have been proposed for DBT are nonreinforced exposure, emotion regulation, attentional control, and decreased rule-governed behavior [37], however, it is not known exactly what is the mechanisms mediating intervention and outcome. The present analysis could suggest that the mechanism of change that has the highest hierarchy is, in fact, *emotional regulation*. Mindfulness, which is one of the central strategies in DBT, impacts the emotional experience by modifying the *automatic response tendencies* associated with emotions. For example when the patient experiences the emotion of anger and intends to fight, the instruction of training is to *observe*, *describe* and *participate* in the emotional experience without acting on it, so mindfulness has the potential to modify the behavioral response and associated thought, in such a way that it has the potential to give the affective experience a different meaning [37]. From a systems perspective, emotion regulation is likely to decrease vulnerability by weakening the strength of the connection between nodes rather than reducing the intensity of emotions (e.g., weakening the association between experiencing a breakup and frantically avoiding abandonment).

In DBT, emotion dysregulation is one of the symptoms reduced in the initial phases of treatment, nevertheless, the connection between treatment and nodes related to identity, could stress the importance that psychotherapy should not focus only on emotional regulation abilities, but also on identity consolidation in the face of diffusion or altered functioning of the self. For DSM 5 (Alternative Model of Personality Disorders) altered functioning in the sense of self (stability of self-image and self-esteem, accuracy of self-appraisal, capacity of emotion tolerance and regulation) has a central role in personality pathology, and is interesting that for this approach, emotional regulation is incorporated in the identity construct [4]. According to the object relations theory, it is proposed that identity disturbance and affective instability arise from a biased representation of self in relation to others, and the use of primitive defenses, such as intense behavioral manifestations of distress [16, 38]. Therefore, treatments which target identity diffusion or altered functioning of the self, such as Mentalization-Based Therapy [39], Transference Focused Psychotherapy [40], Schema-Focused Therapy [22], and Good Psychiatric Management [41], could also be effective to decrease the burden of the disorder.

## Conclusion

Finally, the present work is a direct criticism of the common cause model of psychopathology. Despite the hegemony of the common cause perspective, with some exceptions, the detection of the causes of mental disorders, for example, through genetic methods or brain imaging (e.g., biomarkers), has given unpromising results [42–45]. The case of BPD is not the exception, the empirical evidence does not point to unique causes of the disorder, for example, the psychometric analysis suggests that it is a heterogeneous and inseparable construct from negative affectivity [46]. Also, to date, no genetic or brain BPD etiology has been conclusively identified [44, 47, 48]. Therefore, we suggest that the network approach may be a modern alternative to the common cause model with which research has approached the BPD phenomenon. Despite the positive aspects of the network approximation, this methodology is not free of limitations, and some of these are part of the present investigation. For example, the models implemented in this work (Ising and mixed graphical model) assume that the measurement has no error, which could be unrepresentative of the nature of the self-report data (for example, people could respond randomly and not according to their problem). Likewise, the Ising model requires dichotomous data, although in the present study we used a binarization that reflected those cases that were above the mean in particular symptoms, the practice of dichotomizing a variable of more levels implies loss of information. Future studies could benefit from the use of network models with latent variables [49] or with the development of *in silico* interventions under the assumptions of models that allow ordinal or numerical variables. Another limitation that applies to any group analysis (between-person), such as the one carried out for the *in vivo* intervention, is the assumption that the structure of relationships detected at the group level can be generalized to individuals [50]. By definition, the analyses between-person investigate individual differences that do not vary in a within-person manner on potential repeated measures [51]. Therefore if we were interested, as we are, in explaining intraindividual variation in BPD (e.g., how does the emotional state evolve in a specific person with BPD? How long does it take to return to a baseline emotional state?), studies are needed that carry out intensive longitudinal intra-person measurements. It is possible that considering each individual as a unique system of interacting dynamic processes, we can advance in understanding the disorder so that the treatment for a patient can be personalized in time and place [52].

## Supporting information

S1 Appendix Supplementary table. Stability of all connections and measures of centrality estimated with bootstraping.
(XLSX)

## References

[1] Borsboom D. A network theory of mental disorders. World Psychiatry. 2017;16(1):5–13. doi: 10.1002/wps.20375 28127906 PMC5269502

[2] Fried EI. Studying Mental Health Problems as Systems, Not Syndromes. Current Directions in Psychological Science. 2022; p. 096372142211140. doi: 10.1177/09637214221114089

[3] Borsboom D, Cramer AOJ. Network analysis: An integrative approach to the structure of psychopathology. Annual Review of Clinical Psychology. 2013;9:91–121. doi: 10.1146/annurev-clinpsy-050212-185608 23537483

[4] American Psychiatric Association. Diagnostic and Statistical Manual of Mental Disorders, 5th Edition; 2013.

[5] Cramer A, van Borkulo CD, Giltay EJ, van der Maas HLJ, Kendler KS, Scheffer M, et al. Major Depression as a Complex Dynamic System. PLoS ONE. 2016;11(12):e0167490. doi: 10.1371/journal.pone.0167490 27930698 PMC5145163

[6] Lunansky G, van Borkulo CD, Haslbeck JMB, van der Linden MA, Garay CJ, Etchevers MJ, et al. The Mental Health Ecosystem: Extending Symptom Networks With Risk and Protective Factors. Frontiers in Psychiatry. 2021;12:301. doi: 10.3389/fpsyt.2021.640658 33815173 PMC8012560

[7] Lunansky G, van Borkulo CD, Blanken T, Cramer AOJ, Borsboom D. Bouncing back from life’s perturbations: Formalizing psychological resilience from a complex systems perspective Gabriela Lunansky. Psyarxiv. 2022; p. 1–37. doi: 10.31234/osf.io/ftx4j 39432353

[8] Haslbeck JMB, Ryan O, Robinaugh DJ, Waldorp LJ, Borsboom D. Modeling Psychopathology: From Data Models to Formal Theories. Psychological Methods. 2021;27(6):930–957. doi: 10.1037/met0000303 34735175 PMC10259162

[9] Robinaugh DJ, Haslbeck JMB, Ryan O, Fried EI, Waldorp LJ. Invisible Hands and Fine Calipers: A Call to Use Formal Theory as a Toolkit for Theory Construction. Perspectives on Psychological Science. 2021;16(4):725–743. doi: 10.1177/1745691620974697 33593176 PMC8273080

[10] Borsboom D, van der Maas HLJ, Dalege J, Kievit RA, Haig BD. Theory Construction Methodology: A Practical Framework for Building Theories in Psychology. Perspectives on Psychological Science. 2021;16(4):756–766. doi: 10.1177/1745691620969647 33593167

[11] Richetin J, Preti E, Costantini G, De Panfilis C. The central role of identity in Borderline Personality Disorder: Evidence from network analysis. PLoS ONE. 2017;12(10):e0186695. 29040324 10.1371/journal.pone.0186695 PMC5645155

[12] Southward MW, Cheavens JS. Identifying Core Deficits in a Dimensional Model of Borderline Personality Disorder Features: A Network Analysis. Clinical Psychological Science. 2018;6(5):685–703. doi: 10.1177/2167702618769560 30854263 PMC6402351

[13] Rivnyák A, Pohárnok M, Péley B, Láng A. Identity Diffusion as the Organizing Principle of Borderline Personality Traits in Adolescents—A Non-clinical Study. Frontiers in Psychiatry. 2021;12(July):1–7. doi: 10.3389/fpsyt.2021.683288 34295274 PMC8289896

[14] Peckham AD, Jones P, Snorrason I, Wessman I, Beard C, Björgvinsson T. Age-related differences in borderline personality disorder symptom networks in a transdiagnostic sample. Journal of Affective Disorders. 2020;274(April):508–514. doi: 10.1016/j.jad.2020.05.111 32663983

[15] Linehan MM. Cognitive behavioural therapy of borderline personality disorder. New York: Guilford. 1993;.

[16] Blüml V, Doering S. ICD-11 Personality Disorders: A Psychodynamic Perspective on Personality Functioning. Frontiers in Psychiatry. 2021;12(April):1–8. doi: 10.3389/fpsyt.2021.654026 33935839 PMC8085265

[17] Lunansky G, Naberman J, van Borkulo CD, Chen C, Wang L, Borsboom D. Intervening on psychopathology networks: Evaluating intervention targets through simulations. Methods. 2022;204:29–37. doi: 10.1016/j.ymeth.2021.11.006 34793976

[18] Storebø OJ, Stoffers-Winterling JM, Völlm BA, Kongerslev MT, Mattivi JT, Jørgensen MS, et al. Psychological therapies for people with borderline personality disorder. Cochrane Database of Systematic Reviews. 2020;2020(11). 32368793 10.1002/14651858.CD012955.pub2 PMC7199382

[19] von Klipstein L. The Exploratory Value of Cross-Sectional Partial Correlation Networks: Predicting Relationships Between Change Trajectories in Borderline Personality Disorder; 2021. Available from: 10.34894/NVROJB. PMC8323921 34329316

[20] von Klipstein L, Borsboom D, Arntz A. The exploratory value of cross-sectional partial correlation networks: Predicting relationships between change trajectories in borderline personality disorder. PLoS ONE. 2021;16(7):e0254496. doi: 10.1371/journal.pone.0254496 34329316 PMC8323921

[21] Dickhaut V, Arntz A. Combined group and individual schema therapy for borderline personality disorder: A pilot study. Journal of Behavior Therapy and Experimental Psychiatry. 2014;45(2):242–251. doi: 10.1016/j.jbtep.2013.11.004 24342235

[22] Giesen-Bloo J, van Dyck R, Spinhoven P, van Tilburg W, Dirksen C, van Asselt T, et al. Outpatient Psychotherapy for Borderline Personality Disorder. Archives of General Psychiatry. 2006;63(6):649. doi: 10.1001/archpsyc.63.6.649 16754838

[23] Leppänen V, Hakko H, Sintonen H, Lindeman S. Comparing Effectiveness of Treatments for Borderline Personality Disorder in Communal Mental Health Care: The Oulu BPD Study. Community Mental Health Journal. 2015;52(2):216–227. doi: 10.1007/s10597-015-9866-4 25824852

[24] Wetzelaer P, Farrell J, Evers SM, Jacob GA, Lee CW, Brand O, et al. Design of an international multicentre RCT on group schema therapy for borderline personality disorder. BMC Psychiatry. 2014;14(1). doi: 10.1186/s12888-014-0319-3 25407009 PMC4240856

[25] Giesen-Bloo JH, Wachters LM, Schouten E, Arntz A. The Borderline Personality Disorder Severity Index-IV: Psychometric evaluation and dimensional structure. Personality and Individual Differences. 2010;49(2):136–141. doi: 10.1016/j.paid.2010.03.023

[26] Arntz A, van den Hoorn M, Cornelis J, Verheul R, van den Bosch WMC, de Bie AJHT. Reliability and Validity of the Borderline Personality Disorder Severity Index. Journal of Personality Disorders. 2003;17(1):45–59. doi: 10.1521/pedi.17.1.45.24053 12659546

[27] Pfohl B, Blum N, St John D, McCormick B, Allen J, Black DW. Reliability and Validity of the Borderline Evaluation of Severity Over Time (Best): A Self-Rated Scale to Measure Severity and Change in Persons With Borderline Personality Disorder. Journal of Personality Disorders. 2009;23(3):281–293. doi: 10.1521/pedi.2009.23.3.281 19538082 PMC3608461

[28] Jiménez S, Angeles-Valdez D, Rodríguez-Delgado A, Fresán A, Miranda E, Alcalá-Lozano R, et al. Machine learning detects predictors of symptom severity and impulsivity after dialectical behavior therapy skills training group in borderline personality disorder. Journal of Psychiatric Research. 2022;151:42–49. doi: 10.1016/j.jpsychires.2022.03.063 35447506

[29] van Borkulo C, Epskamp S, Robitzsch wcfA. IsingFit: Fitting Ising Models Using the ELasso Method; 2016. Available from: https://CRAN.R-project.org/package=IsingFit.

[30] van Borkulo CD, Borsboom D, Epskamp S, Blanken TF, Boschloo L, Schoevers RA, et al. A new method for constructing networks from binary data. Scientific Reports. 2014;4(1). doi: 10.1038/srep05918 25082149 PMC4118196

[31] Epskamp S, Borsboom D, Fried EI. Estimating psychological networks and their accuracy: A tutorial paper. Behavior Research Methods. 2017;50(1):195–212. doi: 10.3758/s13428-017-0862-1 PMC5809547 28342071

[32] Marsman M, Borsboom D, Kruis J, Epskamp S, van Bork R, Waldorp LJ, et al. An Introduction to Network Psychometrics: Relating Ising Network Models to Item Response Theory Models. Multivariate Behavioral Research. 2018;53:15–35. doi: 10.1080/00273171.2017.1379379 29111774

[33] Gunderson JG, Stout RL, McGlashan TH, Shea MT, Morey LC, Grilo CM, et al. Ten-year course of borderline personality disorder: Psychopathology and function from the collaborative longitudinal personality disorders study. Archives of General Psychiatry. 2011;68(8):827–837. doi: 10.1001/archgenpsychiatry.2011.37 21464343 PMC3158489

[34] van Nes EH, Scheffer M. Slow Recovery from Perturbations as a Generic Indicator of a Nearby Catastrophic Shift. The American Naturalist. 2007;169(6):738–747. doi: 10.1086/516845 17479460

[35] Linehan MM, Bohus M, Lynch TR. Dialectical Behavior Therapy for Pervasive Emotion Dysregulation: Theoretical and Practical Underpinnings. In: Handbook of emotion regulation. New York, NY, US: The Guilford Press; 2007. p. 581–605.

[36] Díaz JL, Barraza G, Hernández-Fuentes E, Jiménez S. Emotion Words in Spanish: Lexical Selection, Affective Dimensions, Sex and Age Differences. Cognitive Semantics. 2022;8(2):181–209. doi: 10.1163/23526416-08020002

[37] Lynch TR, Chapman AL, Rosenthal MZ, Kuo JR, Linehan MM. Mechanisms of change in dialectical behavior therapy: Theoretical and empirical observations. Journal of Clinical Psychology. 2006;62(4):459–480. doi: 10.1002/jclp.20243 16470714

[38] Clarkin JF, Lenzenweger MF, Yeomans F, Levy KN, Kernberg OF. An object relations model of borderline pathology. Journal of Personality Disorders. 2007;21(5):474–499. doi: 10.1521/pedi.2007.21.5.474 17953502

[39] Bateman A, Fonagy P. Mentalization based treatment for borderline personality disorder. World Psychiatry. 2010;9(1):11–15. doi: 10.1002/j.2051-5545.2010.tb00255.x 20148147 PMC2816926

[40] Doering S, Hörz S, Rentrop M, Fischer-Kern M, Schuster P, Benecke C, et al. Transference-focused psychotherapy v. treatment by community psychotherapists for borderline personality disorder: Randomised controlled trial. British Journal of Psychiatry. 2010;196(5):389–395. doi: 10.1192/bjp.bp.109.070177 20435966

[41] Gunderson J, Masland S, Choi-Kain L. Good psychiatric management: a review. vol. 21. Elsevier; 2018. 10.1016/j.copsyc.2017.12.006 29547739

[42] Lacasse JR, Leo J. Serotonin and depression: A disconnect between the advertisements and the scientific literature. PLoS Medicine. 2005;2:1211–1216. doi: 10.1371/journal.pmed.0020392 16268734 PMC1277931

[43] Adam D. Mental health: On the spectrum. Nature. 2013;496(7446):416–418. doi: 10.1038/496416a 23619674

[44] Borsboom D, Cramer AOJ, Kalis A. Brain disorders? Not really: Why network structures block reductionism in psychopathology research. Behavioral and Brain Sciences. 2019;42. doi: 10.1017/S0140525X17002266 29361992

[45] Tiego J, Martin EA, DeYoung CG, Hagan K, Cooper SE, Pasion R, et al. Precision behavioral phenotyping as a strategy for uncovering the biological correlates of psychopathology. Nature Mental Health. 2023;1:304–315. doi: 10.1038/s44220-023-00057-5 37251494 PMC10210256

[46] Gutiérrez F, Aluja A, Rodríguez JR, Peri JM, Gárriz M, Garcia LF, et al. Borderline, Where Are You? A Psychometric Approach to the Personality Domains in the International Classification of Diseases, 11th Revision (ICD-11). Personality Disorders: Theory, Research, and Treatment. 2022. doi: 10.1037/per0000592 35737563

[47] Leichsenring F, Leibing E, Kruse J, New AS, Leweke F. Borderline personality disorder. The Lancet. 2011;377(9759):74–84. doi: 10.1016/S0140-6736(10)61422-5 21195251

[48] Bohus M, Stoffers-Winterling J, Sharp C, Krause-Utz A, Schmahl C, Lieb K. Borderline personality disorder. The Lancet. 2021;398(10310):1528–1540. doi: 10.1016/S0140-6736(21)00476-1 34688371

[49] Epskamp S, Rhemtulla M, Borsboom D. Generalized Network Psychometrics: Combining Network and Latent Variable Models. Psychometrika. 2017;82(4):904–927. doi: 10.1007/s11336-017-9557-x 28290111

[50] Mcnally RJ. Network Analysis of Psychopathology: Controversies and Challenges. Annual Review of Clinical Psychology. 2021. doi: 10.1146/annurev-clinpsy-081219-092850 33228401

[51] Isvoranu AM, Epskamp S, Waldorp LJ, Borsboom D. Network Psychometrics with R; 2022.

[52] Molenaar PCM. A Manifesto on Psychology as Idiographic Science: Bringing the Person Back Into Scientific Psychology, This Time Forever. Measurement: Interdisciplinary Research and Perspectives. 2004;2(4):201–218. doi: 10.1207/s15366359mea0204_1

